# Behavioral beliefs about genetic counseling among high‐risk Latina breast cancer survivors in Florida and Puerto Rico

**DOI:** 10.1002/cam4.5111

**Published:** 2022-08-08

**Authors:** Jessica N. Rivera Rivera, Claire C. Conley, Eida M. Castro‐Figueroa, Laura Moreno, Julie Dutil, Jennifer D. García, Charité Ricker, Gwendolyn P. Quinn, Hatem Soliman, Susan T. Vadaparampil

**Affiliations:** ^1^ Moffitt Cancer Center Tampa Florida USA; ^2^ Georgetown University Washington District of Columbia USA; ^3^ Ponce Health Sciences University Ponce Puerto Rico USA; ^4^ USC Norris Comprehensive Cancer Center University of Southern California Los Angeles California USA; ^5^ Grossman School of Medicine, Department of OB‐GYN New York New York University New York New York USA

**Keywords:** behavioral beliefs, breast cancer, genetic counseling, Hispanic women, Latina

## Abstract

Compared with non‐Hispanic White women, Latina women are less likely to receive genetic counseling (GC) and testing (GT) following BC diagnosis. This study used secondary data analysis to explore beliefs about GC among Latina BC survivors in and outside the US mainland. GC/GT‐naïve, high‐risk, Spanish‐preferring Latina BC survivors (*n* = 52) in FL and PR completed the Behavioral Beliefs about GC scale. Participants reported high positive beliefs about GC (*M* = 4.19, SD = 0.92); the majority agreed that GC was beneficial to understand cancer risk (90%) and promote discussion (87%) in their family. Participants reported low‐to‐moderate scores for barriers (*M*s = 1.53–3.40; SDs = 0.59–0.90). The most frequently endorsed barriers were desire for additional GC information (*M* = 3.44; SD = 0.90), and GC logistic concerns (*M* = 2.71; SD = 0.80). No statistically significant differences for barriers and benefits scales were identified by place of residence (all *ps* ≥ 0.12). These findings highlight the importance of delivering culturally sensitive GC information to high‐risk Latina BC survivors.

## Graphical abstract

Spanish‐preferring Latina breast cancer survivors had high positive beliefs about genetic counseling and their reported benefits and barriers for genetic counseling were similar for survivors residing in Florida and Puerto Rico. The study findings highlight the factors that should be considered when designing culturally sensitive interventions for Latina breast cancer survivors and suggest that similar intervention contents to promote uptake of genetic counseling could be implemented in Florida and Puerto Rico.

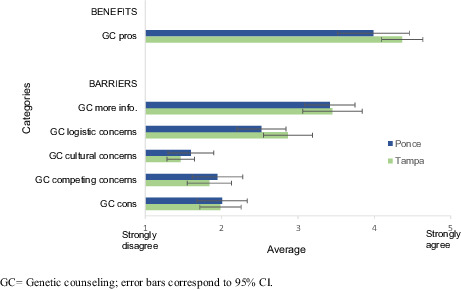

## 1 INTRODUCTION

Genetic counseling (GC) and testing (GT) can inform cancer treatment, facilitate cascade testing, and guide risk management for secondary cancers. Pathogenic variants in the *BRCA1/2* genes are associated with a 55%–72% lifetime risk for breast cancer (BC), 26%–83% for contralateral BC, and 17–59% for ovarian cancer.^1^, ^2^ Despite a similar prevalence of *BRCA1/2* variants^3^, ^4^ and awareness of GC/GT,^5^, ^6^, ^7^ Latina women are less likely to have GT than non‐Hispanic White women.^8^ Structural factors such as relatively few Spanish‐speaking genetic counselors in the mainland US^9^ and the minimal availability of clinics that offer cancer GC in Puerto Rico (PR)^10^ may contribute to this disparity.

Although Latina women's attitudes about GC/GT tend to be positive,^7^, ^11^, ^12^ specific behavioral beliefs (i.e., attitudes about the consequences of GC) are less well studied. The Integrative Model of Behavior Prediction suggests that individuals' demographic and psychosocial characteristics influence behavioral beliefs, which in turn impact health behaviors.^13^ Individuals' exposure to health information can vary by geographical areas, resulting in group beliefs differences. However, to our knowledge, this is the first study exploring if behavioral beliefs about GC differs among high‐risk Latina BC survivors in and outside the US mainland. Identifying potential differences between Spanish‐preferring Latinas in PR versus Florida will be used to develop targeted interventions promoting uptake of GC/GT among Spanish‐speaking Latinas in FL and PR.

## 2 METHODS

### 2.1 Participants and Procedures

This study used secondary analysis of baseline data from a randomized controlled pilot study evaluating a culturally targeted intervention to increase uptake of GC/GT among Latina BC survivors.^14^ All the participants in the intervention group and recruited in PR had access to no‐cost GC/GT during the study or after the study.^14^ Moffitt Cancer Center (IRB #18601) and Ponce Health Sciences University‐Ponce Research Institute (IRB #160607‐EC) Institutional Review Boards approved study procedures. This study was conducted according to the guidelines of the Declaration of Helsinki.

Eligible participants were: (1) female BC survivors; (2) self‐identify as Latina/Hispanic; (3) age ≥25; (4) Spanish‐preferring; (5) GC/GT‐naïve (have not currently scheduled or previously attended GC/GT), and (6) eligible for GC/GT based on National Comprehensive Cancer Network guidelines.^15^ Participants were recruited between January and June 2017 through clinic‐ and community‐based approaches.^11^, ^14^ Interested individuals called the study and a bilingual research assistant screened them for eligibility. A total of 82 women were screened, 80% (*n* = 66) met inclusion criteria, and 79% (*n* = 52) completed the Spanish‐language baseline assessment in‐person, via mail or telephone prior receiving study education about GC/GT. Written informed consent was obtained from all the participants in this study. Participants were compensated with a $40 gift‐card for completing the baseline assessment.

### 2.2 Measures

#### 2.2.1 Sociodemographic and medical characteristics

Included age, race, country of origin, Ashkenazi Jewish ancestry, partner status, education, employment status, income, insurance, years since diagnosis, cancer stage at diagnosis, and cancer treatment.

#### 2.2.2 GC behavioral beliefs

The 30‐item Behavioral Beliefs about *BRCA* GC scale was developed and tested with English and Spanish‐preferring Latinas at‐risk of breast and/or ovarian cancer.^11^ It includes six subscales: (1) “pros” of GC, (2) “cons” of GC; (3) competing concerns; (4) cultural concerns; (5) logistic concerns; and (6) desire for more information (subscale Cronbach's *αs* = 0.61 to 0.95). Items are rated from “strongly disagree” = 1 to “strongly agree” = 5.

### 2.3 Analytic strategy

Chi‐square tests and *t*‐tests examined differences in sociodemographic and medical characteristics by place of residence (FL and PR). Two‐tailed *t*‐tests examined differences in GC behavioral beliefs subscale scores by place of residence. Due to our interest in belief endorsement, reponses to all items assessing behavioral beliefs were collapsed into two categories: (1) has stated belief: “strongly agree” and “agree,” and (2) does not have stated belief: “neither agree nor disagree,” “disagree” and “strongly disagree.”^16^ For each item, two‐tailed chi‐square tests evaluated differences in belief endorsement by place of residence. The most commonly endorsed benefits and barriers across groups were identified. All analyses were conducted using SPSS (version 27, IBM), and αs≤0.05 were considered statistically significant.

## 3 RESULTS

### 3.1 Sample characteristics

Table 1 details participants' (*n* = 52) characteristics. No significant differences were identified by place of residence, with the exception of country of origin.

**TABLE 1:** Sample characteristics by place of residence

Variables	Total (*N* = 52)	Tampa, FL (*n* = 28)	Ponce, PR (*n* = 24)	*p*‐value
Age (M, SD, range)	54.2 (8.8, 32–75)	54.9 (7.0, 40–75)	53.5 (10.7, 32–75)	0.57
Race (*n, %*)				0.29
White	36 (69.2)	20 (71.4)	16 (66.7)	
Black	3 (5.8)	0 (0.0)	3 (12.5)	
Multiple	9 (17.3)	5 (17.9)	4 (16.7)	
Other	3 (5.8)	2 (7.1)	1 (4.2)	
Country of origin (*n, %*)				<0.001^a^
Puerto Rico	29 (55.8)	6 (21.4)	23 (95.8)	
Colombia	11 (21.2)	11 (39.3)	0 (0.0)	
Cuba	9 (17.3)	9 (32.1)	0 (0.0)	
Dominican Republic	1 (1.9)	1 (3.6)	0 (0.0)	
Mexico	1 (1.9)	1 (3.6)	0 (0.0)	
US mainland	1 (1.9)	0 (0.0)	1 (4.2)	
Ashkenazi Jewish ancestry				0.28
Yes	1 (1.9)	0 (0.0)	1 (4.2)	
No	45 (86.5)	24 (85.7)	21 (87.5)	
Do not know	5 (9.6)	4 (14.3)	1 (4.2)	
Partner status (*n, %*)				0.26
Single	3 (5.8)	0 (0.0)	3 (12.5)	
Married/Domestic Partner/Other	34 (65.4)	20 (71.4)	14 (58.3)	
Divorced/Separated	10 (19.2)	5 (17.9)	5 (20.8)	
Widowed	5 (9.6)	3 (10.7)	2 (8.3)	
Education (*n, %*)				0.82
Up to GED/Diploma	16 (30.8)	9 (32.1)	7 (29.2)	
Some college	11 (21.2)	5 (17.9)	6 (25.0)	
College grad or beyond	25 (48.1)	14 (50.0)	11 (45.8)	
Employment status (*n, %*)				0.41
Not employed	20 (38.5)	13 (46.4)	7 (29.2)	
Employed full‐time	16 (30.8)	8 (28.6)	8 (33.3)	
Employed part‐time	9 (17.3)	5 (17.9)	4 (16.7)	
Retired/other	7 (13.5)	2 (7.1)	5 (20.8)	
Household Income (*n, %*)				0.17
<$35,000/year	36 (69.2)	18 (64.3)	18 (75.0)	
≥$35,000/year	14 (26.9)	10 (35.7)	4 (16.7)	
Insurance (*n, %*)				0.20
Private	15 (28.8)	6 (21.4)	9 (37.5)	
Public	31 (59.6)	17 (60.7)	14 (58.3)	
No insurance	6 (11.5)	5 (17.9)	1 (4.2)	
Years since diagnosis (M, SD, range)	6.7 (4.5, 0–24)	7.8 (4.7, 0–24)	5.4 (4.1, 0–18)	0.06
Stage at diagnosis (*n, %*)				0.56
Stage 0	6 (11.5)	2 (7.1)	4 (16.7)	
Stage 1	9 (17.3)	6 (21.4)	3 (12.5)	
Stage 2	12 (23.1)	6 (21.4)	6 (25.0)	
Stage 3	9 (17.3)	6 (21.4)	3 (12.5)	
Stage 4	5 (9.6)	4 (14.3)	1 (4.2)	
Do not know	9 (17.3)	4 (14.3)	5 (20.8)	
Cancer treatment (*n*, %)	52 (100.0)	28 (100.0)	24 (100.0)	
Surgery	49 (94.2)	26 (92.9)	23 (95.8)	0.93
Chemotherapy	41 (78.8)	22 (78.6)	19 (79.17)	0.96
Radiation	30 (57.7)	15 (53.6)	15 (62.5)	0.52
Hormonal therapy	17 (32.7)	9 (32.1)	8 (33.3)	0.54

Abbreviations: M, mean; SD, standard deviation.

^a^ The variable country of origin was dichotomized to Puerto Rico vs other country for Pearson Chi‐Square statistical analysis.

### 3.2 GC behavioral beliefs

**FIGURE 1:**
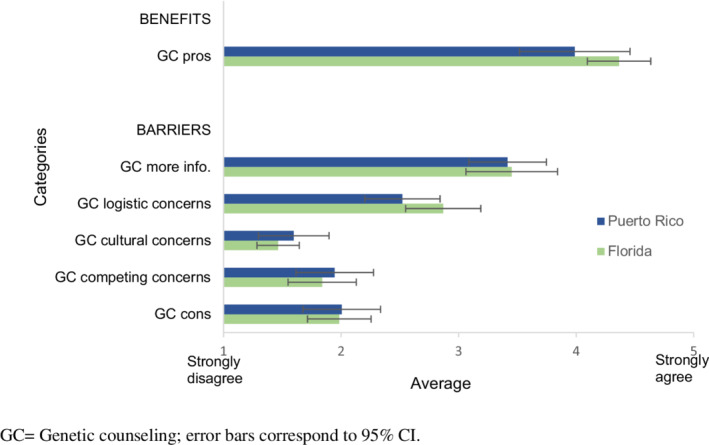
Comparison of benefits and barriers by place of residence.

Participants reported high positive beliefs about GC (*M* = 4.19, SD = 0.92), see Figure 1. Particularly, participants endorsed GC being beneficial for understanding cancer risk of family members (90%, *n* = 47) and for initiating familial discussions about cancer risk (87%, *n* = 45), see Table S1. Participants demonstrated low‐to‐moderate scores on barriers subscales (*M*s = 1.53–3.4; SDs = 0.59–0.90). As presented in Figure 1, frequently endorsed barriers were desire for additional GC information (*M* = 3.44; SD = 0.90), and GC logistic concerns (*M* = 2.71; SD = 0.80). Within the logistic concerns subscale, participants tended to endorse concerns related to insurance coverage and cost, see Table S1. At the subscale level, there were no statistically significant differences by place of residence (all *ps* ≥0.12). Per individual item analyses, only one statistically significant difference (*p* = 0.05) was found; FL participants were more likely to endorse the benefit of GC reducing cancer worry (89%) than those in PR (67%).

## 4 DISCUSSION

The Integrative Model of Behavior Prediction suggests that individuals' demographic and psychosocial factors can influence outcome beliefs for a particular behavior, thereby influencing behavior uptake.^13^ Understanding GC beliefs is relevant to cancer prevention as GC has historically been considered a requisite part of the GT process. To our knowledge this is the first study valuating differences in GC behavioral beliefs for cancer risk by place of residence in the Latinx community. These findings add to the scientific knowledge by suggesting that high‐risk Spanish‐preferring Latina women in FL and PR have similar GC behavioral beliefs. This is consistent with prior research comparing GT knowledge, perceived facilitators, and perceived barriers among Puerto Rican woman living in FL and PR, which did not find any significant differences.^12^ Like prior studies with high‐risk Latinas,^7^, ^11^ our findings suggest that Spanish‐preferring high‐risk Latinas tend to have high positive beliefs about GC. Although participants in our study endorsed benefits of GC, similar to prior studies they wanted more GC information.^11^ Participants' reported lack of adequate information about GC is consistent with previous publications from the parent study, where it was documented that at baseline participants had limited knowledge about GC^14^ and only one third of the participants reported prior provider discussion about GC.^17^ In this study, FL participants more often endorsed GC reduction in cancer worry compared to PR participants, suggesting that Latinas in PR might benefit from further information and clarification about GC, especially on post‐GC decrease in cancer worry.^18^ The observations may also be a consequence of the limited GC resources in PR. Latinas' need for information related to GC was documented as a barrier in this study as well as in prior studies.^7^, ^11^ Furthermore, Latinas' lack of information about risk of jeopardizing insurance and insurance coverage was documented as necessary for GC.

Study findings emphasize the role of *familismo* for GC among Latinas, while other cultural values (i.e., spirituality, fatalism)^19^ were less relevant (for details see Table S1). Previous research has identified *familismo* as an important value for Latina women in the context of GC/GT.^20^, ^21^ In this study almost all the participants believed that GC could benefit their family; however, one third of our sample also perceived that GC would result in worry about a family member developing cancer. Similar results were published in a study of Latinas in New York City,^11^ suggesting that Latinas' GC behavioral beliefs could be similar across different US geographical areas.

Although this study provides relevant and specific information about GC behavioral beliefs among Latina women living in and outside the US mainland, limitations must be considered. First, the small sample size and sample characteristics (i.e., mostly Puerto Ricans and few women representing other Latina populations or Afro‐Latinas, GC/GT‐naïve) may limit the generalizability of the findings. Second, due to convenience sampling, findings might reflect selection bias as individuals with positive attitudes about GC/GT might be more likely to participate. Finally, responses of those who completed in‐person or by telephone surveys might have been more influenced by social desirability bias compared to those completed by mail.

Identification of GC behavioral beliefs differences and similarities across different geographical areas are crucial when adapting interventions to increase use of genetic services. Our findings suggest that the content of the information presented in the interventions targeting uptake of GC among Latinas women in FL and PR could be similar, even when they are from different countries of origin. Based on our findings, these interventions should include a general GC education and focus on *familismo* as an important value.

## AUTHOR CONTRIBUTIONS

Jessica N. Rivera Rivera: formal analysis, methodology, visualization, writing ‐ original draft, and writing ‐ review and editing. Claire C. Conley: conceptualization, data curation, methodology, visualization, writing ‐ original draft, and writing ‐ review and editing. Eida M. Castro‐Figueroa: conceptualization, funding acquisition, writing ‐ review and editing. Laura Moreno: investigation, data curation, and writing ‐ review and editing. Julie Dutil: conceptualization, funding acquisition, data curation, and writing ‐ review and editing. Jennifer D. García: investigation, project administration, and writing ‐ review and editing. Charité Ricker: resources, writing ‐ review and editing. Gwendolyn P. Quinn: conceptualization, funding acquisition, writing ‐ review and editing. Hatem Soliman: resources and writing ‐ review and editing. Susan T. Vadaparampil: conceptualization, formal analysis, funding acquisition, investigation, methodology, project administration, resources, supervision, visualization, writing ‐ original draft, and writing ‐ review and editing.

## FUNDING INFORMATION

Biostatistics Core Facility at the H. Lee Moffitt Cancer Center & Research Institute, a National Cancer Institute‐designated Comprehensive Cancer Center (P30CA076292; PI: Cleveland); the National Cancer Institute (U54CA163071, PIs: Matta & Dutil; U54CA163068, PIs: Wright & Monteiro; T32CA090314, PIs: Brandon & Vadaparampil).

## CONFLICTS OF INTEREST

All authors declare no conflicts of interest.

## ETHICS STATEMENT

All procedures performed in studies involving human participants were in accordance with the ethical standards of the institutional and/or national research committee and with the 1964 Helsinki declaration and its later amendments or comparable ethical standards. All procedures were approved on 2/25/2016 by the Moffitt Cancer Center (IRB #18601) and on 6/29/2016 by Ponce Health Sciences University‐Ponce Research Institute (IRB #160607‐EC) Institutional Review Boards. Informed consent was obtained from all individual participants included in the study.

## Supporting information


Table S1

## Data Availability

De‐identified data from this study will be made available by emailing the corresponding author as allowed by institutional IRB standards.
