# Supplementary material for: Behavioral beliefs about genetic counseling among high‐risk Latina breast cancer survivors in Florida and Puerto Rico
Source: Cancer Med. 2022 Aug 8;12(4):4701–6. doi: 10.1002/cam4.5111 (PMC9972095; doi:10.1002/cam4.5111)
Supplement: Supplementary file 1 — Table S1 [file CAM4-12-4701-s001.docx]

Supplemental Table 1. Perceived benefits and barriers for GC among high-risk Latina breast cancer survivors.

| **Items** | **% Agree (n)** |
| --- | --- |
| *Benefits* |  |
| Genetic counseling would help me better understand cancer risks of other family members. | 90.4 (47) |
| Genetic counseling would help me initiate discussions about cancer risk in my family. | 86.5 (45) |
| I believe there are important benefits to getting genetic counseling even if I decide not to undergo genetic testing for breast and/or ovarian cancer. | 84.6 (44) |
| Genetic counseling would help me decide whether I should undergo genetic testing for breast and/or ovarian cancer. | 82.7 (43) |
| Genetic counseling would reduce my fear and concerns about developing (or having a recurrence of) breast or ovarian cancer. | 78.8 (41) |
| Genetic counseling would help me decide whether to undergo preventive surgery. | 75.0 (39) |
| Genetic counseling would help me make important life decisions (e.g., having children). | 68.6 (35) |
| *Barriers* |  |
| I need to get more information about what genetic counseling has to offer. | 73.1 (38) |
| I feel like I need more information about the ways I could benefit from genetic counseling for breast and/or ovarian cancer. | 71.2 (37) |
| I would be interested in getting genetic counseling, but I want to know that my health insurance would not be jeopardized or put in danger. | 50.0 (26) |
| I would be interested in getting genetic counseling, but I first want to be sure my insurance would cover the cost | 46.2 (24) |
| I worry that my health insurance would not cover the cost of genetic counseling | 40.4 (21) |
| I would be interested in getting genetic counseling, but I am unsure of where to go to get counseling (e.g., don’t know where to go, who to speak with to make an appointment) | 34.6 (18) |
| Genetic counseling would make me worry about the breast and/or ovarian cancer risk of other family members (e.g., mother, daughters). | 34.6 (18) |
| To benefit from genetic counseling, I would need to have a better background (more schooling) in science. | 32.7 (17) |
| Getting genetic counseling is a low priority on my list of things to do. | 15.4 (8) |
| Getting genetic counseling would be too expensive for me | 11.5 (6) |
| It would be distressing for me to talk to a genetic counselor. | 7.7 (4) |
| Genetic counseling would not help me deal with my fears and uncertainty about developing (or having a recurrence of) breast and/or ovarian cancer. | 7.7 (4) |
| Getting genetic counseling would take up too much of my time | 3.8 (2) |
| Undergoing genetic counseling could jeopardize my health insurance. | 3.8 (2) |
| Genetic counseling would not provide me with any means of preventing breast and/or ovarian cancer. | 3.8 (2) |
| I am too busy taking care of my children (or other family members) to get genetic counseling at this time | 3.8 (2) |
| I have too many other things to worry about compared to getting genetic counseling. | 1.9 (1) |
| Getting genetic counseling is not consistent with my religious or spiritual beliefs | 1.9 (1) |
| I feel I already know my chances of getting breast or ovarian cancer, so I wouldn’t learn anything more from genetic counseling. | 0 (0) |
| My doctor (or nurse) has spoken with me about my risk of breast and/or ovarian cancer, so I wouldn’t learn anything new from genetic counseling | 0 (0) |
| I am more concerned about the health of other family members compared to my own, so getting genetic counseling is not of interest to me right now | 0 (0) |
| I believe that if I am diagnosed with breast and/or ovarian cancer I will die (*fatalismo*), so there is no purpose for genetic counseling | 0 (0) |
| I believe that if I am diagnosed with breast and/or ovarian cancer it is my destiny (*destino*), so there is no purpose for getting genetic counseling | 0 (0) |
